# Electromagnetic Field Assessment as a Smart City Service: The SmartSantander Use-Case

**DOI:** 10.3390/s17061250

**Published:** 2017-05-31

**Authors:** Luis Diez, Ramón Agüero, Luis Muñoz

**Affiliations:** Engineering Communications Department, University of Cantabria, Santander 39005, Spain; ramon@tlmat.unican.es (R.A.); luis@tlmat.unican.es (L.M.)

**Keywords:** EMF exposure, smart city, sensors, dosimetry

## Abstract

Despite the increasing presence of wireless communications in everyday life, there exist some voices raising concerns about their adverse effects. One particularly relevant example is the potential impact of the electromagnetic field they induce on the population’s health. Traditionally, very specialized methods and devices (dosimetry) have been used to assess the strength of the E-field, with the main objective of checking whether it respects the corresponding regulations. In this paper, we propose a complete novel approach, which exploits the functionality leveraged by a smart city platform. We deploy a number of measuring probes, integrated as sensing devices, to carry out a characterization embracing large areas, as well as long periods of time. This unique platform has been active for more than one year, generating a vast amount of information. We process such information, and the obtained results validate the whole methodology. In addition, we discuss the variation of the E-field caused by cellular networks, considering additional information, such as usage statistics. Finally, we establish the exposure that can be attributed to the base stations within the scenario under analysis.

## 1. Introduction

The presence of wireless cellular communications in everyday life is unquestionable. Furthermore, current forecasts predict this becoming even more relevant in the forthcoming years [1]. However, there also exist some concerns about the potential adverse effects of such technologies. One of the most controversial ones is the possible impact of the Electromagnetic Fields (EMF) they induce on humans’ health. Some people have expressed their doubts about this particular aspect.

In fact, all European countries have some regulations that limit the EMF generated by wireless base stations. In order to monitor that these are respected, the most widespread methods use dedicated measuring devices, called dosimeters, in order to assess whether the field caused by a base station is within the corresponding limits. These devices are rather expensive, and their use is usually punctual, so that they are not exploited to carry out characterizations during longer periods of time.

In this paper, we present a different and novel approach, which was one of the main outcomes of the LEXNET collaborative research project [2]. The proposed method makes use of a large number of low-complexity (and low-cost) devices, which are integrated into the SmartSantander testbed [3], to continuously monitor the EMF during long periods of time. The combination of these devices and the smart city deployment leverages a unique platform, which has been, at the time of writing, taking measurements for a time period spanning over a year.

The main contributions of this paper are: (1) the description of the overall testbed, discussing how the low-complexity dosimeters exploit the facilities provided by the smart city platform; (2) the evaluation of the values that have been gathered during more than one year and their impact on the human exposure to EMF.

The paper is structured as follows. Section 2 discusses the LEXNET project’s overall approach, as well as some other related initiatives, showing the differences with the proposed platform and methodology. Section 3 describes the testbed that has been deployed, by integrating the low-complexity dosimeters into a smart city platform. The characterization of the EMF field during a long period of time, as well as the exposure induced on the population are depicted in Section 4. Finally, Section 5 concludes the paper, highlighting its most relevant results and some aspects that might be tackled by exploiting the available testbed.

## 2. Preliminaries

As was mentioned earlier, the testbed that is presented in this paper was one of the most relevant outcomes of the LEXNET European project. The overall objective of the project was to define techniques and solutions to reduce the exposure to electromagnetic fields induced by wireless communications, without jeopardizing the Quality of Experience (QoE) perceived by end users. In order to do so, a novel metric, the so-called Exposure Index (EI), was designed. It was conceived of so as to account for the exposure suffered by the population, considering different usage patterns, types of users, periods of the day and other Information and Communication Technologies’ (ICT) statistics. Below, we introduce a simplified version of such a metric and also discuss an initiative that shares some of the characteristics of the platform that we are discussing in this paper, highlighting their differences.

### 2.1. The LEXNET Exposure Index

The design of the LEXNET Exposure Index had to face some contradicting goals. On the one hand, it should be a reasonably simple parameter, so that it could be easily incorporated into network management procedures. On the other hand, it should be also able to combine a large number of information elements of different nature. In order to tackle these objectives, the EI is defined to estimate the average exposure of a population during a certain time interval, in a given area, considering both downlink and uplink communications. Since the influence of the electromagnetic fields strongly depends on various factors (age, gender, posture, etc.), an averaging procedure was proposed to derive a value that adequately considers the whole population. Finally, it is worth mentioning that the EI only accounts for the exposure induced by cellular wireless communication systems (as well as WiFi), leaving aside other technologies, such as radio and television broadcasting services, which are less flexible in terms of their management.

The EI is defined as a summation of exposure components, which in turn correspond to a particular configuration, defined by a segment of population under certain circumstances when using a particular technology. A simplified version of the exposure index is defined in Equation (1) [4].

(1) $$EI^{SAR}\left[\frac{W}{Kg}\right]=\frac{1}{T}\sum_{t}^{N_{T}}\sum_{p}^{N_{p}}\sum_{r}^{N_{RAT}}\sum_{e}^{N_{E}}\sum_{u}^{N_{U}}f_{t,p,r,e,u}\cdot \left[\sum_{u}^{N_{u}}\left(d^{UL_{t,p,r,e,u}}\cdot {\bar{P}}_{TX}\right)+d^{DL_{t,p,r,e,u}}{\bar{S}}_{RX}\right]$$

where $d^{UL}$ and $d^{DL}$ are normalized raw dose values for the uplink and downlink respectively, multiplied by the time spent in each specific configuration. These are expressed as a normalized value of the Specific Absorption Rate (SAR), corresponding to a particular configuration, multiplied by the time users spend at such configuration. Hence, *f* represents the population fraction corresponding to such a configuration. Finally, ${\bar{P}}_{TX}$ and ${\bar{S}}_{RX}$ are the average transmitted power and incident power density, respectively, again for each of the considered configurations. Furthermore, the parameters used for establishing the different configurations are enumerated below:

- $N_{T}$ is the number of considered periods, for instance day or night.
- $N_{P}$ corresponds to the amount of different population categories in the area.
- $N_{RAT}$ indicates the different Radio Access Technologies (RATs) used by the end-users.
- $N_{E}$ accounts for the number of environments, such as urban, sub-urban or rural.
- $N_{U}$ represents the number of different usage profiles [5], which consider various parameters, such as the posture, traffic load, etc.

As can be seen, the EI combines a relatively large number of aspects. Hence, depending on the stakeholder using it, different aspects might be given a stronger relevance. Network operators willing to exploit it for management operations might consider different possibilities. For instance, techniques that are able to reduce the transmission power in a dynamic way would have an impact on the uplink exposure and just for those periods of time when the user is actually having an ongoing communication. On the other hand, planning strategies are more focused on the downlink exposure, induced by the base stations. Precisely, the platform we are discussing in this paper focuses on the downlink contribution of the EI, and the results might be therefore exploited to broaden current network planning procedures. On the other hand, authorities (e.g., town councils), which have no control over either the network or devices, might monitor downlink component of the EI, by measuring the power density, to ensure that the values do not exceed certain limits.

### 2.2. Related Work

**Figure 1:**
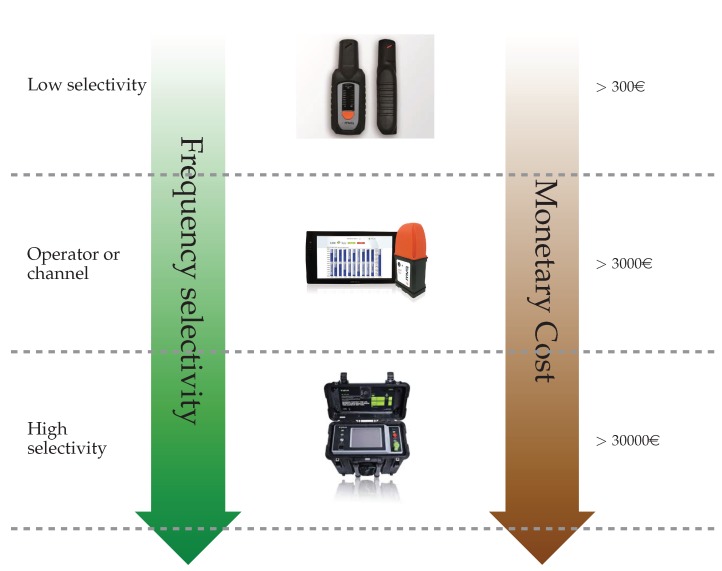
Rough comparison of devices according to their capabilities and prices.

As was said earlier, EMF is traditionally characterized by means of punctual measurement campaigns, which are carried out with rather expensive and dedicated devices. This approach has the advantage that it can yield rather accurate measurements. However, it cannot be used to carry out broad characterizations, considering large areas and/or long periods of time. The platform we introduce in this paper fosters a rather different methodology. It is based on the deployment of a large number of measuring devices, which are continuously taking measurements, on top of a smart city testbed. In this sense, we can assess the variations of the EMF throughout a day (considering different time intervals) or during different periods (working days, weekends, holidays, etc.). This novel approach brings about a much broader assessment both in temporal and spatial terms, although some precision is lost, due to the simplification of the devices, which guarantees that a higher density deployment could be afforded. We can thus conclude that the two approaches are in fact complementary. Figure 1 presents an overall comparison of different types of commercial EMF sensing devices according to their capabilities and cost. As can be observed, devices that provide frequency selectivity are relatively expensive for a massive deployment. As for the the device used in our deployment, it was able to differentiate between the most relevant frequency bands with a rough cost of 300€. This combination made it quite unique, compared to existing solutions, and brought the possibility of using a dosimeter as the IoT device. In fact, although some accuracy could eventually be lost, using the IoT infrastructure enables embedding continuous EMF monitoring as another smart city service. This can actually facilitate its adoptions by relevant stakeholders (for instance, local authorities), since they are already used to other similar services, brought by the generic purpose smart city framework.

However, there are some initiatives that are worth mentioning, since they share some of the main characteristics of the platform discussed herewith. One of them is a deployment fostered by the Government of Catalonia, which comprises more than 300 fixed probes, 22 of them placed in the city of Barcelona. The monitoring equipment assesses the EMF at different frequencies, and measurements have been taken since 2013. The observed values can be consulted in [6]. The main objective of the project was to assess that the measured values were respecting the legal limits. As a consequence, they have focused on particularly sensitive places, such as kindergartens, schools or hospitals, and the corresponding density is therefore much lower. In addition, the deployed devices (according to the data that can be seen in the available web page) do not distinguish between different bands, providing a single value, which corresponds to a rather wide spectrum. Hence, it would quite likely include the exposure induced by other wireless services, such as radio and television broadcasting. As will be seen later, the information provided by our platform differentiates between four bands, and temporary variations, within different intervals, can be easily seen.

Another initiative aiming to provide continuous EMF monitoring is the one proposed by the Hermes project [7], which provides both wide and narrow band measurements. While this solution might provide a functionality similar to ours, it does not exploit a smart city infrastructure, but relies on dedicated devices (as was the case of the Catalonia network), thus making the deployment and data management more complicated. Likewise, the moniT [8] and SEMONT [9] projects also proposed solutions to foster a continuous monitoring, also promoting ad hoc deployments with dedicated devices. It is also worth highlighting the work reported in [10], where the authors exploited a wireless mesh network to deploy sensor devices for continuous EMF monitoring. The proposed system was based on a dedicated testbed, specifically deployed for this purpose, and featured more advanced measuring devices (Spectran Analyzer, HF 6060, Aaronia AG, Strickscheid , Germany), more expensive (around 1000€) than the ones we integrated in our platform. Hence, the feasibility of this solution to cover wide areas might be questioned. As can be seen, none of the mentioned papers exploited a generic-purpose smart city platform to deploy E-field probes as if they were a regular sensing device, as was done in the solution presented herewith.

Another important difference between these testbeds and the one discussed in the paper is that their devices are dedicated, specific probes, which take measurements and send them to a control center. Hence, their reconfiguration might likely require a manual intervention. We fully exploit the functionality of a generic smart city platform, where the low-complexity dosimeters are seen as regular IoT sensing devices, so that they can be remotely managed (e.g., reprogrammed) by the control operations brought by the platform.

All in all, the smart city integration offers three main advantages: (1) the devices are much less expensive, since they do not need to provide the means to send the information to a remote location, a functionality that is taken care of by the smart city platform; (2) all of the functionalities of the platform (data storage, data mining, external access, etc.) can be straightforwardly used to process the information gathered by the dosimeters; (3) some configuration parameters of the probes, such as the measuring frequency, can be remotely reprogrammed.

## 3. EMF Testbed

This section discusses the platform that was deployed so as to characterize the EMF exposure within a relatively broad area. In particular, we focus on the Santander (Northern Spanish city) downtown, where 40 dosimeters were deployed. The testbed leverages the functionalities provided by the SmartSantander platform to build up a measurement tool, which in this particular case is made of a large number of dosimeters. This macro tool aims to provide two main functionalities, related to the calculation of the EI: (1) covering large-scale scenarios; (2) performing continuous measurements during long periods of time (i.e., years).

Although it overcomes the limitations of traditional dosimeter-based approaches, it also presents some drawbacks. In particular, it is initially limited to the assessment of downlink EMF, due to the particular positioning of devices, mostly attached to lampposts and urban furniture. In this sense, the large distances to end-users would prevent the devices from obtaining near-field measurements, which are required to characterize uplink exposure.

We start by describing the smart city testbed where the dosimeters were integrated, as well as the design and basic operation of these sensors. Afterwards, we discuss some of the characteristics of the area under analysis, since they would have an impact on the observed results, as well as the methodology that was used for the deployment. Finally, the main outcomes of the measurement tool are depicted, illustrating the whole deployment and the web portal that was created to access the data [11]. It is worth mentioning that, at the time of writing, there is public access to this portal, as well as to the historical data gathered by the platform.

### 3.1. SmartSantander

SmartSantander, the smart city testbed, in which the low-complexity dosimeters are integrated, was conceived of to satisfy various requirements from different stakeholders, from researchers to service providers, including utility providers and local authorities. Its deployment started during a European project, but it has been extended and broadened ever since. At the time of writing (April 2017), the platform consists of approximately 3000 IoT nodes, which are deployed following a three-tier architecture, based on their logical role. The platform is open and can be accessed in [12]. The aforementioned three levels are briefly depicted below:

- IoT node: This is the entity that actually performs data acquisition. Most of them are fully integrated within a repeater, which provides the required communication capabilities, while others are deployed standalone and connect to repeaters by means of proprietary communications, which usually require wireless transmissions (for instance, ferro-magnetic sensors that are buried and provide smart-parking information).
- Repeaters: They provide the first communication level, creating a multihop capillary network that gathers the information provided by the sensing devices. The repeaters are usually deployed on walls or lampposts, and they all create a multi-hop network, based on the 802.15.4 technology. All repeaters are grouped into 25 clusters, yielding a star topology.
- Gateways: They receive the information from the repeaters and connect the capillary network with the SmartSantander backbone, where the information is stored and processed.

In order to deploy devices for new services, they must be first appropriately registered within the platform, by means of a logical identifier (node ID), 802.15.4 parameters (operating channel and gateway) and link-level security setup (the communication from the repeater to the gateway is ciphered). Once a device is deployed and connected to the testbed, the registration procedure is automatically performed, by mapping it to its logical identifier. For that, an Authentication, Authorization and Accounting (AAA) process is used, ensuring: (1) that only information coming from nodes registered within a cluster is accepted; (2) the correct identification of the information coming from a particular device, since all of the packets from the same sensing node are tagged with its unique identifier; and (3) the appropriate registration of context metadata.

The low-complexity dosimeter is seen, from the SmartSantander platform perspective, as a new type of sensing device, providing some information (i.e., electrical field), which is enriched with the following metadata: geographical location, temporal stamp and measurement units. The dosimeters were attached to a repeater, responsible for controlling the measurement process. In particular, it controls the sensing process and is appropriately configured to interact with the platform as described above.

### 3.2. Low Complexity Dosimeter

**Figure 2:**
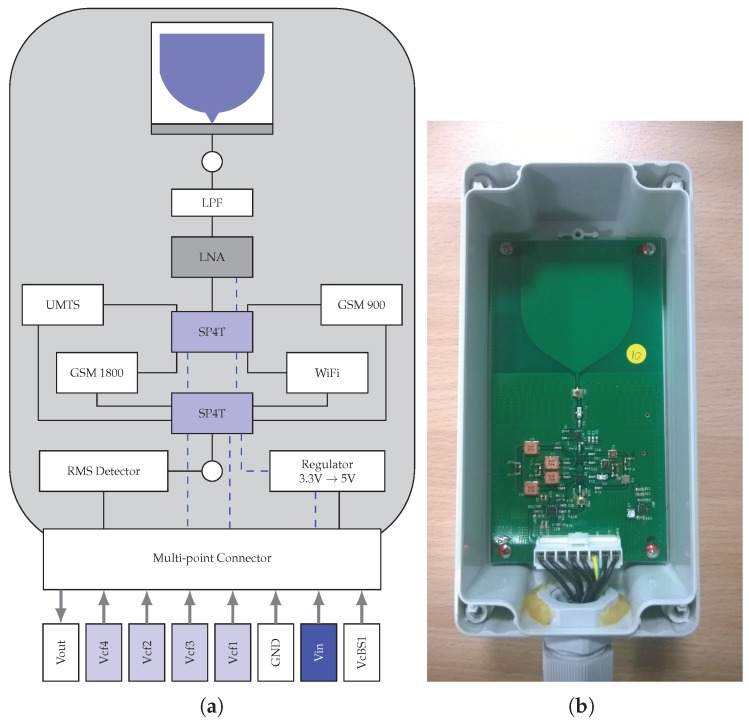
Dosimeter design and implementation. (**a**) Dosimeter block diagram; (**b**) dosimeter prototype.

A novel approach was followed during the design of the low-complexity dosimeter, with the main goal of enabling massive deployments. There was a trade-off, since it lacks the accuracy of more advanced devices. The result was a probe able to obtain E-field samples in different frequency bands. The device had a relatively low cost, compared to traditional dosimeters, and included the corresponding logic to facilitate its integration with the smart city testbed. Figure 2 illustrates the block diagram of the dosimeter and shows a photograph of the device that was finally deployed. For a more detailed description of the dosimeter design, the reader can refer to [13]. The probe was installed together with a dedicated repeater, which takes care of its control while reading the corresponding E-field measurements. Once the data are obtained from the probe, they are forwarded through the capillary network to the gateways.

When the device was designed, we analyzed the usage of the different wireless communication bands in the city of Santander. As a consequence, it was decided that the dosimeter should be able to monitor the frequencies shown in Table 1. They correspond to the most relevant cellular bands, covering 2G (typically in 900 and 1800 MHz), 3G (2.1 GHz) and 2.4 GHz, which is the most widespread Industrial Scientific and Medical (ISM) frequency. When the design was done (beginning of 2014), LTE technology was not using dedicated frequencies, but the operators were exploiting other bands to carry such communications (refarming), as could be seen during an extensive measurement campaign that was performed [14]. The dosimeter (being low cost) is not able to differentiate between the technologies within the same band, but their presence shall be taken into account when estimating the EI based on the EMF measurements.

As can be seen in Figure 2, the dosimeter uses a printed monopole antenna. Since it is a single polarized probe, it eases the integration with the rest of electronics components within a PCB, thus helping to lessen the corresponding cost. However, this solution requires an appropriate estimation of the isotropic EMF from the mono-polarized measurements. In order to calibrate the process, extensive off-line measurements with more capable devices were carried out, obtaining the penalty factors to be applied to the dosimeter measurements. Furthermore, additional operational parameters, such as sensitivity and dynamic range, were defined according to several studies’ findings [15,16,17], which showed that the E-field from wireless communication systems is usually below 1 V/m. All in all, the low-complexity dosimeter offered a 5-mV/m sensitivity, with a range that reached up to 5 V/m.

Regarding the integration with the SmartSantander testbed, the device was designed to be externally supplied, in order to reduce its overall cost and to avoid hindering the RF measurements. In this sense and depending on its particular location within the smart city testbed, the dosimeter could be supplied either by the repeater controlling it or directly from lampposts. Furthermore, the device includes a number of digital inputs that can be managed, for instance to select the frequency band and to enable the whole measurement process, thus allowing its control from the repeaters.

### 3.3. Scenario Description and Deployment Methodology

**Figure 3:**
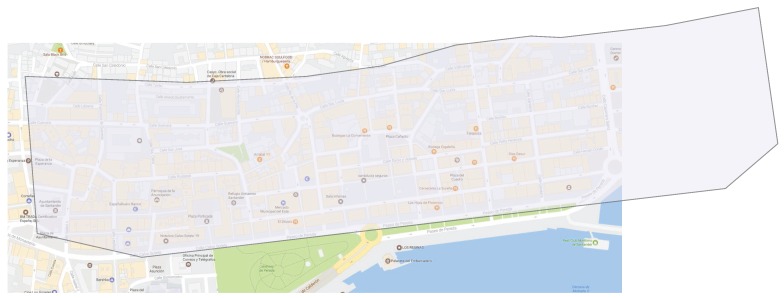
Target scenario in the downtown of Santander city.

Forty low complexity dosimeters were deployed to cover a surface of approximately $0.5\;{\mathrm{km}}^{2}$. As can be seen in Figure 3, the scenario comprises both wide avenues in an open area, facing the sea, as well as narrower streets, with mid-height buildings. In order to determine the position of the low-complexity dosimeters, we also took into account the location of the base stations within the scenario. The deployment strategy [18] had a two-fold objective: (1) to ensure an accurate assessment of the E-field statistical distribution; and (2) to develop an interpolation model, so that we could estimate the EMF at any point, based on the deployed probes.

Hence, we started by carrying out a thorough simulation-based study, exploiting a precise 3D model (with a resolution of 5 m) of the area under analysis. The Volcano [19] coverage-based simulation platform [20] was used to characterize the EMF. Based on its results, we were able to establish the dosimeter density that might be needed. The process was to randomly deploy a varying number of dosimeters and compare the EMF that was estimated based on their measurements with the real one.

**Figure 4:**
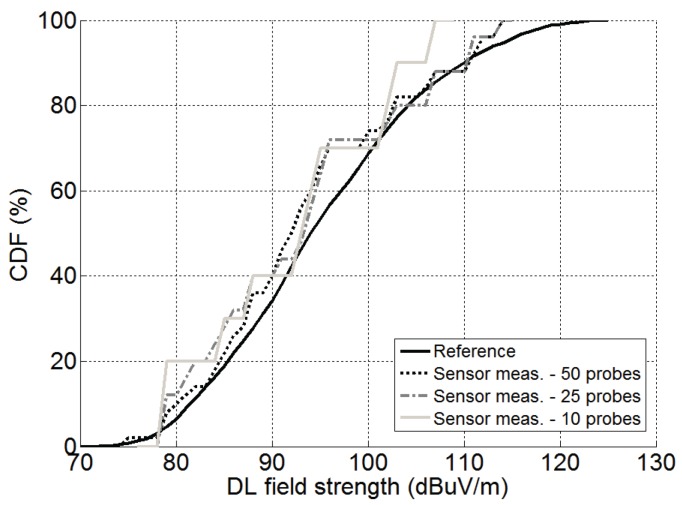
Simulated E-field strength CDF from dosimeter measurements. Figure 6 from [18].

Figure 4 shows the Cumulative Distribution Function (CDF) of the E-field strength for three probe-densities, namely 10, 25 and 50 probes. The CDF is the probability that the E-field would take a value lower than or equal to the corresponding abscissas coordinates. We can see that the estimated EMF is closer to the real one for higher probe densities, although the difference is not particularly relevant, being slightly higher for stronger EMF values. Furthermore, Table 2 shows the average values of the Root Mean Square Error (RMSE), as well as the mean error (bias). We can see that the gain when increasing from 10–25 is quite relevant (≈40%), but this is much less noticeable when going from 25–50. The main conclusion that can be drawn is that the 40 devices that were deployed in the area under analysis are enough to provide an accurate characterization.

The second step was to establish the exact locations to deploy the dosimeters. This was done in an iterative process. At every step, based on the values measured until then, an algorithm was applied to optimally find the locations of the next device, to improve the interpolation model. In particular, the algorithm consists of two clashing criteria to carry out measurements in uncharted areas (exploration) and densify the number of dosimeters in those areas that are found to be more interesting (exploitation), such as those having fast E-field variation, or showing strong differences with their close-by area. The algorithm gives each location a certain score, as a result of the trade-off between exploration and exploitation. These scores are used to sort the locations where the next dosimeters should be deployed. In [18], we provided more detailed information about this procedure.

Since the ultimate goal of the deployment was not only to measure the EMF, but to assess the exposure index, it becomes relevant to understand how the networks are used within the area under analysis. We recall that the low-complexity dosimeter has limited capabilities, and it cannot differentiate between technologies, but it only provides the E-field for each of the considered bands (as a whole). In addition, when the measurements were made, we could not match one single technology with every band, since operators are known to use spectrum refarming techniques, deploying technologies in frequency bands initially assigned to others. This is specially relevant for the initial roll-out phases of 4G-LTE networks, which can easily make use of some of the cellular bands that we are monitoring.

**Figure 5:**
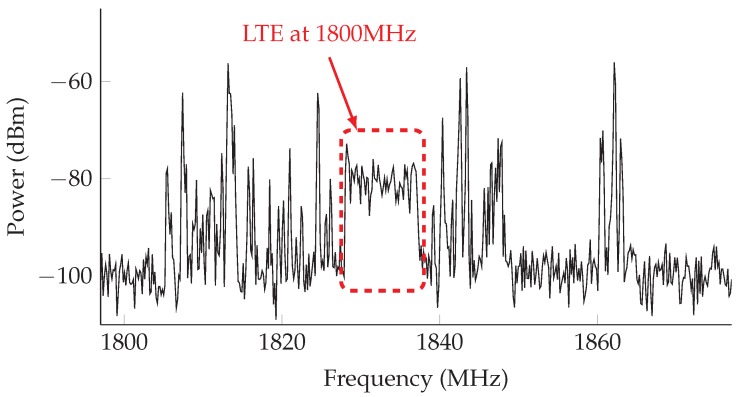
Operator refarming at 1800 MHz. Figure 4b from [14].

In order to address this, a preliminary characterization of the target area [14] was performed using a spectrum analyzer. We learned that the active operators were indeed exploiting refarming techniques, as can be seen in Figure 5, which shows the spectrum for the 1800-MHz band. The figure clearly illustrates the presence of LTE signal, within the GSM-based communications (higher peaks).

### 3.4. Web Portal

**Figure 6:**
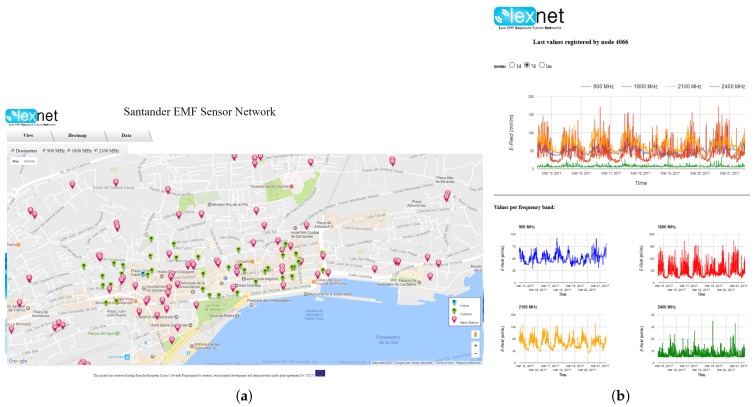
Web portal: deployment map and E-Field variation at a single dosimeter. (**a**) LEXNET deployment; (**b**) E-field variation.

Once the deployment was completed, we made the gathered information publicly available by means of a web portal, to facilitate data acquisition and visualization. Figure 6 depicts the main view of the portal, where users can see the deployment of the dosimeters (i.e., their position), differentiating whether they have been installed indoors or outdoors.

Besides the dosimeter location, the web interface also shows the position of base stations within the area; see Figure 6a. These access elements can be filtered by their frequency band, and a link is provided for each of them, offering further pieces of information, in particular E-field measurements in its vicinity when they were installed. This context information can be afterwards used to better understand the measurements gathered by the dosimeters. Furthermore, quick data visualization can be also obtained from each dosimeter, as shown in Figure 6b. This preliminary visualization can be used to observe how the parameter varies, as well as to roughly compare the values provided by different dosimeters. In this sense, it could also be used as a means to assess the validity of the whole measurement methodology or to identify failing probes.

**Figure 7:**
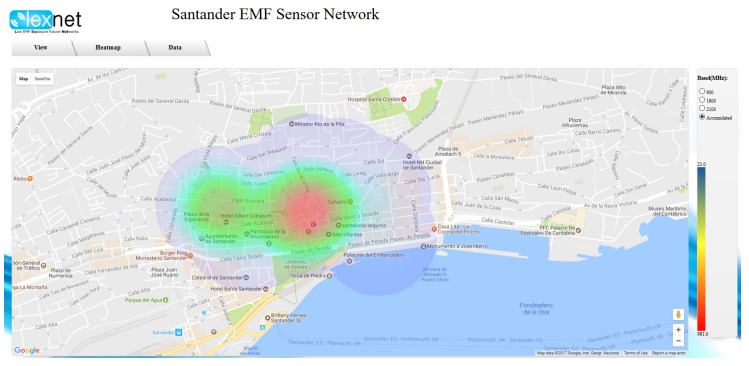
Heat map of the EMF exposure on Santander downtown.

As previously mentioned, one of the main objectives of the measurement tool is the assessment of the EMF exposure over large areas. While this can be performed by analyzing the data provided by the dosimeters, the web portal also gives an overall representation of exposure throughout the target area, by means of a dynamic heat map, as is shown in Figure 7. The heat map illustrates the evolution of the EMF measurements during the last 24 h, filtered by the corresponding frequency band or considering the accumulated exposure. While the information provided by the map is clearly insufficient for a thorough EMF analysis, it allows one to quickly identify potential hot-spots in the city, as well as the temporal evolution of the E-field.

**Figure 8:**
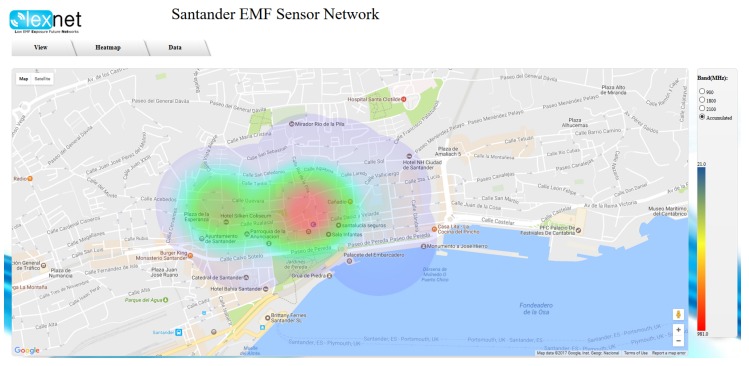
Dosimeter selection for data retrieval.

Finally, the web portal can be also be used to graphically retrieve the data from the different devices, by simply clicking the selected device, as illustrated in Figure 8. We can use different time-frames (day or week) and filter the information in terms of the frequency bands. In addition, more detailed filters can be applied, by selecting the day periods reflecting the usage patterns in Spain (working hours, breaks, rest, etc.). The information can be also downloaded, in simple text files, which might be used by anybody interested in the E-field induced by real cellular network deployments in exploitation.

## 4. EMF Exposure Evaluation

After depicting the deployed testbed, this section discusses some of the main results that have been obtained by means of this measurement framework, in terms of both E-field characterization and exposure index computation within the Santander downtown area. First, Section 4.1 describes the methodology that has been used to compute the EI from both the E-field measurements and life-segmentation statistics. Then, a thorough evaluation of the E-field, from information gathered during more than one year within the target area, will be discussed in Section 4.2. Special attention is paid to the suitability of these measurements for the EI calculation. Finally, the downlink component of the EI will be also evaluated.

### 4.1. EI Computation Methodology

As was mentioned earlier, the EI definition considers not only electro-magnetic field values, but also a range of data elements that are connected to life segmentation, use patterns of communications devices and SAR values. This information has been calculated for a typical reference scenario, which was defined within the LEXNET project and whose main characteristics are shown in Table 3. All of them are based on measurements and simulation-based studies that were carried on during the project lifetime.

As can be seen, the population is assumed to belong to four different segments, each of them with four traffic profiles: none, light, moderate and heavy. It is worth highlighting that users within the none-traffic profile are also considered, since they would also be impacted by the E-field exposure (passive receivers). The traffic profiles are different for every population segment and radio access technology. The reader may refer to [5] for a succinct discussion of these values and the assumptions that were made to obtain them.

According to the EI definition that was described in Section 2, the corresponding contribution from each configuration can be defined as a scaled value of either the mean transmission power (${\bar{P}}_{tx}$) or the mean received power density (${\bar{S}}_{rx}$) for uplink and downlink exposure, respectively. In this sense, we can simplify the EI as follows:

$$\begin{array}{cc} EI^{SAR}\left[\frac{W}{Kg}\right] & =\frac{1}{T}\sum_{t}^{N_{T}}\sum_{p}^{N_{p}}\sum_{e}^{N_{E}}\sum_{u}^{N_{U}}f_{t,p,e,u}\cdot \left[\sum_{u}^{N_{u}}\left(d^{UL}\cdot {\bar{P}}_{TX}\right)+d^{DL}{\bar{S}}_{RX}\right]\\ EI^{SAR}\left[\frac{W}{Kg}\right] & =\frac{1}{T}\sum_{i}^{I}\left(K_{UL}^{i}\cdot {\bar{P}}_{TX}\cdot K_{DL}^{i}+{\bar{S}}_{RX}\right) \end{array}$$

where $K^{i}$ are constant exposure coefficients for a specific configuration and are calculated by scaling a reference Whole Body averaged SAR (WBSAR) to account for ICT usage and technology. The value of the WBSAR is typically used as a basic constraint for human exposure to radio frequency [21]. This coefficient-based simplification was proposed in [5] to facilitate the EI computation. The reference values of the WBSAR were estimated by means of studies conducted in different European countries, assuming a transmission power and a power density of 1W and 1W/m^2^, for uplink and downlink, respectively. Besides, WBSAR reference values were calculated for different services (e.g., voice, data, etc.) and both in outdoor and indoor conditions. Finally, the coefficients to calculate the EI were obtained by modulating the WBSAR reference values with ICT usage statistical information.

For instance, the coefficient of the EI uplink contribution from indoor voice calls during morning periods using 3G networks, and assuming a reference time interval of one day, would be thus defined as:

$$K_{UL}^{3G/indoor/voice/day}=\frac{{\%}_{3G_{connections}}}{24\cdot 3600}\cdot \left[t_{morning/indoor/voice}\cdot WBSAR_{3G/indoor/voice}^{UL}\cdot {\%}_{users_{voice}}\right]$$

where the factors $WBSAR_{3G/UL/indoor/voice}$ represent reference values for this particular configuration. In addition, the use of this formulation to calculate the EI from the values provided by the low-complexity dosimeter platform requires some further simplifications to be made. As discussed before, the measurements gathered by the dosimeters do not provide meaningful values for the uplink exposure, and we should just thus consider downlink EI components. Besides, since the low complexity dosimeter is not able to differentiate between technologies within the same frequency band, the average whole band WBSAR has been estimated, by using typical usage values (i.e., how the band is used by the various cellular technologies). Finally, we will focus on the outdoors EMF exposure, since we did not deploy dosimeters in indoor environments. All in all, Table 4 shows the values of these parameters, for the different technologies and population segments in the considered urban scenario [5].

Afterwards, and before the EI calculation can be carried out, the raw information gathered by the low-complexity dosimeters should be processed, to take into account uncertainty sources and particular scenario characteristics.

As was already mentioned, the low complexity dosimeter has a mono-axial probe, and correction factors need therefore to be applied to yield an accuracy level comparable to that from an isotropic device. Furthermore, the low-complexity dosimeters take wide-band measurements, not being able to distinguish the different technologies that are deployed by the existing operators within the same frequency band. Last, but not least, the installation of devices might also cause some inaccuracy, due to the existing urban furniture, which has to be considered during the data process.

**Figure 9:**
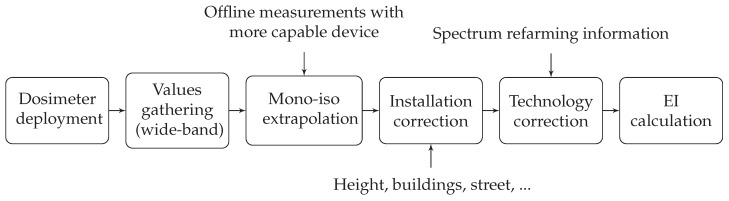
Exposure Index (EI) calculation in a smart city scenario.

Figure 9 illustrates the whole data processing methodology. Once the E-field values are obtained, they are scaled with the mono-axial to isotropic and installation correction factors, whose values can be found in Table 5.

These values were obtained by carrying out on-field measurements, in Santander city, with more capable devices (advanced spectrum analyzers). This campaign embraced static measurements at different locations, so as to compare them with the E-field values gathered by the low-complexity devices. We covered the whole downtown of the city (area where the platform is deployed) to validate the overall methodology and the correct values provided by the dosimeters. The reader may refer to [22] for a more thorough description of the measurement campaign.

Afterwards, the contribution of the different technologies within each of the frequency bands is estimated, based on ICT statistics. We have to recall here that the usage and population factors that are applied in the EI computation is not based on frequency bands, but on the technologies. Once all of the correction operations are applied, the E-field values are transformed into power density, and the EI is finally calculated by applying the WBSAR factors shown above.

### 4.2. E-Field Characterization and Exposure Index

This section discusses some of the results that have been obtained by using the measurement platform and methodology that have been previously described. As has been already said, their main target is to carry out the EMF exposure characterization within large areas during long time periods. We will first study the evolution of E-field measurements, discussing how this parameter evolves. Afterwards, we compute the downlink component of the exposure. In order to do so, we have used all of the data gathered for a complete year (2016), focusing on the outdoors E-field and leaving aside the ISM 2.4-GHz band, since the obtained values were much lower than for the cellular technology bands.

**Figure 10:**
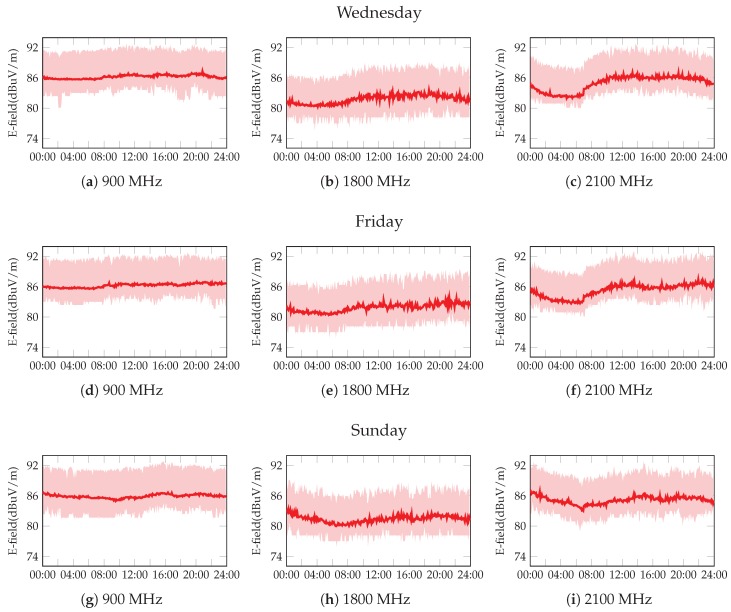
Average value of the E-field during a day; each point is obtained every five minutes. Upper and lower values represent the confidence interval.

For the first group of results, we select a single dosimeter, and we study the temporal variation of the E-field values. This analysis aims to validate the 24-h usage, as the reference time period *T* for the calculation of the EI. Figure 10 illustrates such temporal E-field evolution. We select a particular dosimeter and three days: Wednesday, Friday and Sunday. For a single day, we have datasets of 288 samples, since the E-Field is measured every 5 min. Then, we take the 52 datasets during the year (one per week), and we plot both the average value, as well as the 95% confidence interval. Each of the nine figures is thus obtained by processing ≈15,000 samples. We can see that there is a non-negligible variance of the results, although the trend is quite stable. The E-field values for the 1800-MHz band are the lowest ones, which corresponds to the fact that this is the least used band. We can also see that the 3G band (2100 MHz) shows a more relevant variation on the E-field, which is due to the stronger impact of traffic patterns over CDMA-based technologies. Note that this is the unique band without GSM technology. Another interesting conclusion can be derived by comparing the evolution of different days. In this sense, the E-field on Saturday nights (left part of the lower figures) is clearly higher than in the other days. This reflects the fact that the downtown has several restaurants and pubs and people usually go out on Saturday nights.

We have afterwards studied the statistical behavior of the E-field during different day periods. In particular, we have defined five time intervals, according to different activity levels and habits in Spain:

- Standard Working Time (SWT): comprises the time periods in the ranges 9:00–13:00 (a.m.) and 16:00–19:00 (p.m.)
- Rest Period (RP): is assumed to be in the ranges 14:00–16:00 (a.m.) and 19:00–22:00 (p.m.)
- Night: comprises the time interval when communications are less likely, comprising from 23:00 (p.m.) to 7:00 (a.m.)

**Figure 11:**
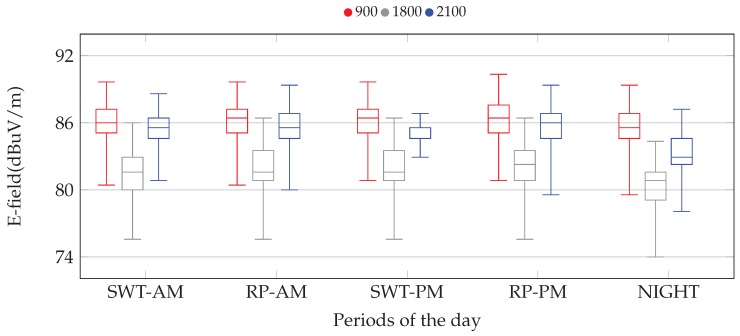
Distribution of the E-field for different frequency bands and during different periods of time. The results are grouped for the different times of the day (standard working time and resting periods), and the values for the different frequency bands are indicated in each group.

Based on such classification, Figure 11 depicts the statistical distribution of the E-field during different day periods for one particular dosimeter, using a box-plot diagram. For each period of the day and frequency band, the boxes indicate the 25 (Q1), 50 (Q2 or median) and 75 (Q3) percentiles. Furthermore, based on Q3 and Q1, we establish the interquartile range (IQR), as IQR=Q3−Q1, so that the bottom/top lines in the graphs are defined as Q1−1.5·IQR and Q3+1.5·IQR, respectively.

As was also observed before, these results yield a rather high variability for all frequency bands. Furthermore, there is not a clear impact of the day time over the E-field values, but for a slight increase during the evening rest period. Besides, the results also show a more noticeable E-field reduction during night hours.

**Figure 12:**
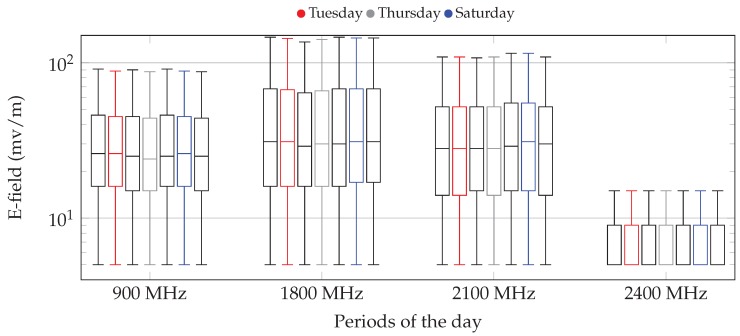
Distribution of the E-field for different frequency bands and days of the week. The results are grouped for the frequency band, and the days of the week are highlighted in each group.

Once we have seen how the E-field varies during a day, Figure 12 shows the distribution of values taken by a single dosimeter, for the seven days of a week. The statistical distribution is represented by using box-plot diagrams, whose values have the same meaning as in Figure 11.

As was the case of the previous results, we are considering the values taken during a whole year, 2016. We can again see that there is not a clear difference between the E-field measured for each day. The graphs yield a non-negligible increase for the 900-MHz band during the weekend. However, for the 1800-MHz band, the E-field on Sundays gets lower, being rather constant for the rest of the week. This is due to the fact that during most of the year, this band has been exploited by the operators to carry 4G traffic (refarming), as was previously discussed. We cannot see any clear pattern for the 2100-MHz band, which is mostly devoted to 3G technology.

Hence, we can conclude that the measurement variability at a particular point is rather low, validating our initial assumption of using a 24-h interval as the reference period for EI calculation. As can been seen, we ensure not overlooking EMF peaks and valleys during the day, for the different frequency bands. It is worth pointing out that the same behavior was indeed observed in the remaining dosimeters, and the conclusion could be therefore generalized to the whole set of points of the deployment.

**Figure 13:**
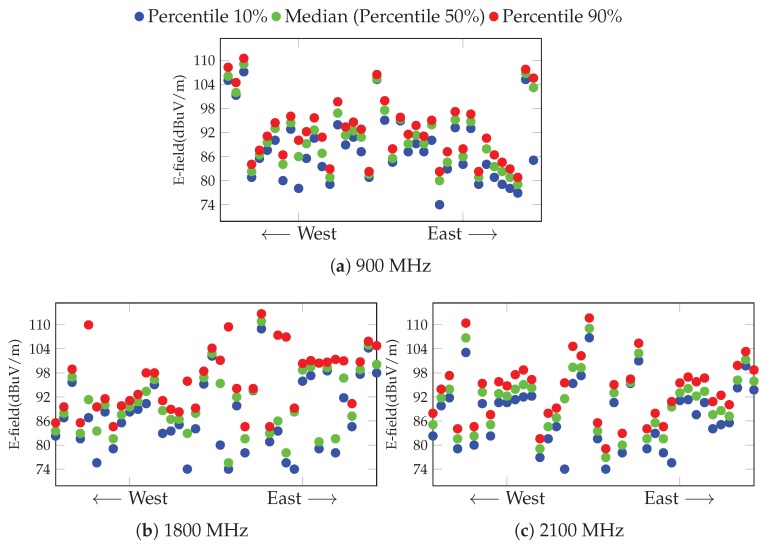
Spatial diversity of measurement among the dosimeters for each frequency band. Each point represents a percentile of the values obtained by one dosimeter during the year 2016. The X-axis indicates the geographical location of the dosimeter, so that the lower the *x*-value, the more east the location is.

Once we have seen how the E-field varies at a single measurement point, Figure 13 shows the correlation between the values provided by close-by dosimeters. We sort them according to their longitude, from the most western dosimeter (the one which is more at the left on Figure 6a) towards the most eastern one. We plot, for each of the 40 devices, three percentile values of the E-field ($\Pi_{r},\;r=0.1,0.5,0.9$), which represent the value below which there is an *r* probability of the E-field. These results yield interesting insights on how the E-field varies within the scenario under study. On the one hand, the measurements taken by a dosimeter do not show a high variability, yielding a tight distribution, where the highest and lowest percentile values are quite close to each other, thus confirming what was previously seen. On the other hand, although there is a clear correlation between some of the close-by devices, there are some cases where the measurements exhibit larger differences. This somehow corroborates the initial assumption that the density of probes needs to be high enough to leverage an accurate characterization. Both aspects are observed for the three frequency bands. This is in fact confirming the simulation-based analysis presented in Section 3.3.

In order to derive the EI from the E-field values provided by the dosimeters, we will use the methodology that was previously discussed. As a first step, we need to identify the technologies that are used in each frequency band, so as to apply the appropriate factors; see Table 4. This is complicated by the fact that operators exploit refarming techniques and make use of some bands to deploy technologies for which they were not initially conceived. Hence, we need to take some assumptions regarding the spectrum usage within the scenario. In this sense, based on the outcome of a more accurate measurement campaign, carried out with a Received Signal Strength Indicator (RSSI) scanner, we extracted the following technology distribution: 2G technologies (i.e., GSM) is using both 900- and 1800-MHz bands; 3G networks are allocated in the 2100-MHz band; and 4G is only deployed in the 1800-MHz band. Hence, there are two different technologies (2G and 4G) coexisting in the same band (1800 MHz). Based on field measurements, (see Appendix 2.8 in [22]), we assume that the E-field induced by 2G is 4.5-times higher than that caused by LTE. As a consequence, we establish the corresponding correction factors for GSM and LTE: 0.18 and 0.81, respectively.

**Figure 14:**
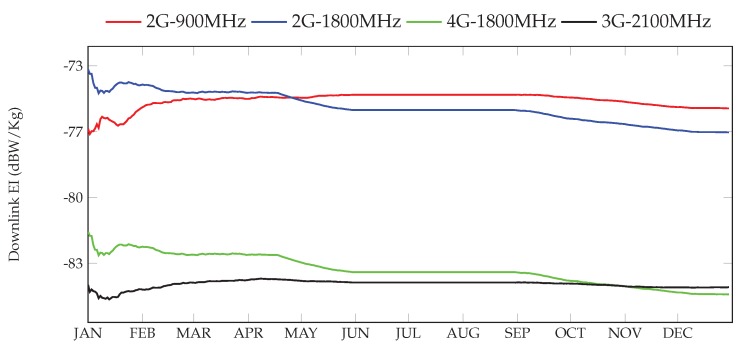
Temporal evolution of the downlink component of the EI in Santander.

Figure 14 shows the temporal evolution of the downlink EI, considering the contribution coming from the different technologies. It can be clearly seen that the strongest contribution is the one caused by 2G technology, which is almost 10 dB higher than the one observed for 3G and 4G technologies. Besides, we can also observe, for the two 2G bands, a smooth decrease of the EI, being slightly more relevant for the 1800-MHz case. This might be caused by the fact that during the last few months, cellular operators have deployed 4G networks using white-spaces left by TV broadcasting services (800 MHz). In this sense, it is sensible to assume that the E-field values can be affected by adjacent bands due to the sensitivity of the dosimeters. On the other hand, the values observed for the 2100-MHz band, uniquely devoted to 3G, shows a rather constant level during the whole year.

## 5. Conclusions

This paper has introduced a novel methodology to characterize the electromagnetic field induced by wireless networks. As opposed to the more traditional dosimetry-based approach, which usually relies on punctual measurements carried out with dedicated and expensive devices, we exploit the functionalities of a smart city platform to deploy a number of low-complexity probes. We have shown that we can indeed tackle the characterization of large areas, considering long periods of time. We have analyzed the information gathered during a whole year (during which the platform has been active), and the obtained results validate the overall approach. We have seen that there are no remarkable variations on the values measured at a single location. In addition, although the results showed some correlation between close-by dosimeters, we also saw that there might be relevant differences. The high-density of probes is precisely addressing this issue. Last, we have exploited additional pieces of information (usage statistics and detailed measurements) to estimate the exposure induced by the different wireless communication technologies. We have seen that the impact of GSM is quite relevant, as compared with 3G and 4G networks.

The deployed testbed is active and can be publicly accessed through a web portal. In this sense, its data are fully available to the scientific community, which might use real E-field values induced by cellular networks in the exploitation for various purposes. For instance, it would bring about a novel key performance indicator to be considered during the planning phase of a wireless network.

## Figures and Tables

**Table 1:** Frequency bands monitored by the low-complexity dosimeter.

Typical Application	3GPP Band	Freq. Band (MHz)
GSM 900 downlink	8	925–960
GSM 1800 downlink	3	1805–1880
UMTSdownlink	1	2110–2170
WiFi 2.4 GHz	–	2400–2483.5

**Table 2:** Error statistics from different dosimeter deployments [18].

50 Probes		25 Probes		10 Probes	
Mean Bias (dB)	RMSE (dB)	Mean Bias (dB)	RMSE (dB)	Mean Bias (dB)	RMSE (dB)
1.38	2.52	1.4	2.63	1.71	3.87

**Table 3:** Reference scenario for life segmentation data [5].

Parameter	Values
Time periods	Day, Night
Population segments	Child (<15), Young (15–29), Adult (30–59), Senior (>59)
Traffic load	None, Light, Moderate, Heavy
Environment	Outdoor, Indoor, Commuting
RAT	2G, 3G, 4G, WiFi

**Table 4:** Outdoor average Whole Body SAR (WBSAR) according to population segment and technology.

Technology	Factor	Children (13.9%)	Young (32.8%)	Adults (28.2%)	Seniors (15.1%)
Urban 2G	$WBSAR_{day}$	52.92	31.2	32.76	45.24
	$WBSAR_{night}$	7.56	15.6	17.16	17.16
Urban 3G	$WBSAR_{day}$	48.51	28.2	29.61	40.89
	$WBSAR_{night}$	6.93	14.1	15.51	15.51
Urban 4G	$WBSAR_{day}$	45.36	25.2	26.46	36.54
	$WBSAR_{night}$	6.48	12.6	13.86	13.86
WiFi	$WBSAR_{day}$	45.36	25.2	26.46	36.54
	$WBSAR_{night}$	6.48	12.6	13.86	13.86

**Table 5:** Correction factors to be applied to the E-field samples.

**Band (MHz)**	900	1800	2100	2400
**Mono-Axial to Isotropic**	0.74	0.66	0.24	0.23
**Installation**	6.20	2.9	1.16	2.97

## Abbreviations

The following abbreviations are used in this manuscript:

EMF	Electromagnetic Fields
LEXNET	Low-EMF Exposure Networks
QoE	Quality of Experience
EI	Exposure Index
IoT	Internet of Things
SAR	Specific Absorption Rate
RAT	Radio Access Technology
AAA	Authentication, Authorization and Accounting
RF	Radio Frequency
CDF	Cumulative Distribution Function
ISM	Industrial, Scientific and Medical
LTE	Long Term Evolution
PCB	Printed Circuit Board
RMSE	Root Mean Square Error
GSM	Global System for Mobile communications
WBSAR	Whole averaged Body SAR
ICT	Information and Communication Technology
SWT	Standard Working Time
RP	Rest Period
IQR	Interquartile Range
